# Validation of an Accelerometer Sensor-Based Collar for Monitoring Grazing and Rumination Behaviours in Grazing Dairy Cows

**DOI:** 10.3390/ani11092724

**Published:** 2021-09-17

**Authors:** Muhammad Wasim Iqbal, Ina Draganova, Patrick C. H. Morel, Stephen T. Morris

**Affiliations:** School of Agriculture and Environment, College of Sciences, Massey University, Private Bag 11-222, Palmerston North 4442, New Zealand; I.Draganova@massey.ac.nz (I.D.); P.C.Morel@massey.ac.nz (P.C.H.M.); S.T.Morris@massey.ac.nz (S.T.M.)

**Keywords:** AfiCollar, accelerometer sensor, grazing dairy cows, grazing behaviour, rumination behaviour

## Abstract

**Simple Summary:**

Grazing behaviour measures herbage intake and varies according to herbage type, climate conditions, and social status of the animal within the herd. Rumination behaviour indicates the digestive efficiency and health status of the animal and varies according to herbage quality, type, and maturity. Both intake and digestive efficiency substantially affect the animal’s performance. Knowledge about grazing and rumination behaviours could improve the health and welfare of animals, pasture management, and overall production efficiency in a grazing-based system. Dairy farming in New Zealand is characterised by pasture-based grazing systems. In this system, monitoring the individual animal behaviour, especially grazing and rumination, has been less explored, primarily because visual observation is labour intensive and subject to human error. Advancements in precision livestock farming (PLF) have introduced behaviour monitoring tools that can record grazing and rumination behaviours. However, the adoption of PLF is still a challenge in the farming community due to the lack of research-based knowledge to address the accuracy of PLF tools in a grazing-based system. The objective of this study was to evaluate the accuracy of a grazing and rumination behaviour monitoring device (AfiCollar) for grazing dairy cows.

**Abstract:**

This study evaluated the accuracy of a sensor-based device (AfiCollar) to automatically monitor and record grazing and rumination behaviours of grazing dairy cows on a real-time basis. Multiparous spring-calved dairy cows (*n* = 48) wearing the AfiCollar were selected for the visual observation of their grazing and rumination behaviours. The total observation period was 36 days, divided into four recording periods performed at different times of the year, using 12 cows in each period. Each recording period consisted of nine daily observation sessions (three days a week for three consecutive weeks). A continuous behaviour monitoring protocol was followed to visually observe four cows at a time for each daily observation session, from 9:00 a.m. to 5:00 p.m. Overall, 144 observations were collected and the data were presented as behaviour activity per daily observation session. The behaviours visually observed were also recorded through an automated AfiCollar device on a real-time basis over the observation period. Automatic recordings and visual observations were compared with each other using Pearson’s correlation coefficient (r), Concordance correlation coefficient (CCC), and linear regression. Compared to visual observation (VO), AfiCollar (AC) showed slightly higher (10%) grazing time and lower (4%) rumination time. AC results and VO results had strong associations with each other for grazing time (r = 0.91, CCC = 0.71) and rumination time (r = 0.89, CCC = 0.80). Regression analysis showed a significant linear relationship between AC and VO for grazing time (R^2^ = 0.83, *p* < 0.05) and rumination time (R^2^ = 0.78, *p* < 0.05). The relative prediction error (RPE) values for grazing time and rumination time were 0.17 and 0.40, respectively. Overall, the results indicated that AfiCollar is a reliable device to accurately monitor and record grazing and rumination behaviours of grazing dairy cows, although, some minor improvements can be made in algorithm calibrations to further improve its accuracy.

## 1. Introduction

Grazing and rumination are predominant behaviour activities in cattle, in terms of the daily allocated time, as cows, on average, spend 90–95% of their daily time grazing, ruminating, and resting [1]. Grazing is the natural feed intake behaviour in cows and chewing of cud (rumination) is the most important activity after grazing [2]. The grazing behaviour of cows affects their ability to consume the optimal quantity of herbage [3]; hence, influencing their milk production [4]. Moreover, it has been suggested by previous studies that dry matter intake (DMI) from grazed herbage is regulated by the time spent grazing and intake rate [5,6,7]. Similarly, rumination has been associated with nutrition and health in dairy cows [8,9] Thus, measuring grazing and rumination behaviours can be a potential management tool to facilitate improved health, welfare, and productivity in dairy cows [10,11]. Measuring those behaviours is also potentially important for the management of pasture and feed availability [12].

In previous decades, measuring behaviour mostly relied on visual observation or video recording, whereas, those methods are very time-consuming, labour intensive, and subject to human error [13,14]. Therefore, an automated system for monitoring the behaviour of dairy cows became an important requirement for dairy production systems. That need paved the path for new approaches to develop an automated system to resolve the problem. Owing to the advancements in precision livestock farming (PLF), various sensors monitoring grazing and rumination behaviours have been manufactured over the past two decades. Those PLF tools are easy to handle, and have less labour input and human interference [15]. The first automated system to record jaw movements was developed for sheep, which consisted of a silicon tube packed with carbon granules and could be fitted over the muzzle of the animal [16]. The system was further improved by later research to enable differentiation between mastication chews and prehension bites [17]. Subsequently, a microcomputer-based system using a sensor with a digital recorder was initially developed [18] and later upgraded [19,20]. The sensors, currently available, can potentially distinguish jaw movements, and respective algorithms exist to interpret them into chews and bites [21]. Data recorded using these sensors have been reported with 94% accuracy to record grazing time and rumination time [22].

There are four types of behaviour monitoring sensors available—voltage detecting noseband pressure sensors, sound detecting acoustic sensors, motion-detecting accelerometer sensors, and electrical signals detecting electromyographic sensors. The potential disadvantage in pressure and acoustic sensors are: tightening of the halter on individual animals generates different pressure values in pressure sensors, and disturbance by environmental noise leads to misclassifications of behaviour signals by acoustic sensors [15]. Therefore, considering more recent accelerometer sensors is more practical in an outdoor-based farm. An accelerometer sensor is a type of electronic device able to convert physical acceleration, such as motion or gravity into waveform as an output signal. It can detect and calculate both static (due to gravity) and dynamic (due to animal’s movements) accelerations, as well as a low-frequency component of the acceleration [23,24]. A 1-axis accelerometer sensor was first presented to monitor eating and ruminating behaviours in cattle [25]. Technology was further improved and the 3-axis (x, y, z) accelerometer sensor was used subsequently to monitor the eating behaviour of cows on a pasture [26]. Accelerometer sensors offer 90% functional accuracy [15], but few recent studies have focused on the evaluation and application of accelerometer sensors to measure grazing and rumination behaviours [27,28].

AfiCollar (AC), developed by Afimilk Ltd. Kibbutz Afikim 1514800, Israel, is an automated device used to monitor and record eating and rumination behaviours on a real-time basis. The AfiCollar device is equipped with a triaxial accelerometer sensor allowing the identification and classification of eating and rumination behaviours based on the patterns of the animal’s head movements [29]. The collar has a built-in integrated algorithm to calculate and classify eating time and rumination time by using the raw data. The behaviour data are transmitted to a wireless-based farm station once the animal wearing AfiCollar comes under the range of the base station. The data are then manually downloaded from the computer attached to the base station. The main advantages of the collar device are its robust mechanical design and its ability to continuously monitor and record individual animal behaviour for several months; moreover, it operates with almost no human interference.

The AfiCollar device has been tested for measuring eating and rumination behaviours in the indoor dairy production system [30]. The collar device has not been validated for grazing dairy cows. The primary objective of this study was to validate the collar device by evaluating its accuracy to monitor and record grazing and rumination behaviours of dairy cows with voluntary movements in a pasture grazing condition.

## 2. Materials and Methods

### 2.1. Experimental Animals and Their Diet

A group of multiparous spring-calved lactating dairy cows (*n* = 48) fitted with AfiCollar devices were selected for the visual observation of their grazing and rumination behaviours. The cows were kept in a grazing-based system on a New Zealand-based farm. The cows included in the study had a 16.3 ± 4.4 L per day milk yield, 465 ± 54 kg body weight, and 4.6 ± 0.4 body condition score. Breeds of the cows were Jersey, Holstein-Friesian, and KiwiCross (a crossbreed of Holstein-Friesian and Jersey). The cows were in different lactations (1, 2, 3), and were milked once a day at 6:00 a.m. The cows were fed on pasture mainly consisting of ryegrass (*Lolium perenne*) mixed with red clover (*Trifolium pratense*) and white clover (*Trifolium repens*), and allocated a dry matter intake of ~20 kg per cow per day. The cows were kept in the same grazing paddock (2.08 ha) for 24 h (except milking time from 6:00 to 8:00 a.m.), and had ad libitum access to a water supply.

### 2.2. Behaviour Observation

Specific behaviour activities of cows observed were grazing (cow actively looking for grass while walking with the head down, including biting and chewing pasture) and rumination (cow starts to regurgitate the chewed bolus in the mouth for re-mastication and ends once the bolus is swallowed), as defined in a previous study [31].

The total period of valid observation was 36 days divided into 4 recording periods that were conducted at different times of the year (Table 1) to cover the whole lactation period. At the time of each recording period, cows were at a different stage of lactation (different days in milk). Days in milk are the number of days for a cow since calving. Each recording period was allocated a sequence of 9 continuous daily observation sessions. Each observation session consisted of 8 h per day, from 9:00 a.m. to 5:00 p.m. to cover maximum daylight hours. Each observation session was performed for three consecutive weeks with three consecutive days per week. Out of the selected 48 cows, 12 cows were included in each recording period. A set of 4 cows was observed at a time for 3 consecutive daily observation sessions per week with a set of 4 other cows for the next daily observation sessions for the next week and so on. The observation was performed by a single trained observer following a continuous behaviour recording protocol [3]. The cows being observed were the only cows in the grazing paddock, and observed by the same observer throughout the observation period [32,33].

The cows were observed by the observer from a distance of ~30 m. Four time-synchronised stopwatches were used (one for each cow) to record grazing time and rumination time in the form of minutes spent per hour (min/h) by the cow on specific behaviour activity. At the start of each behaviour activity, a stopwatch was started to run the timer counting the minutes spent on that behaviour activity. The timer remained running until the behaviour activity was stopped or switched to a different behaviour activity. The timer was paused when the cow paused the behaviour activity or went to drink water for example. The stopwatches were reset once the hour ended and started again for the next consecutive hour. Minute-per-hour spent on grazing and rumination were obtained separately for each hour. The per-hour grazing time and rumination time per cow for eight hours were totalled to calculate grazing time and rumination time per daily observation session. The data collected through visual observation were presented as grazing time and rumination time per daily observation session, and manually stored in a Microsoft Excel spreadsheet (Microsoft Excel, version 2016).

### 2.3. Sensor-Based Collar for Behaviour Monitoring

The automated AfiCollar device consisted of a proprietary 3D (x, y, z) accelerometer sensor fitted within a box and positioned on the right side of the animal’s neck. The accelerometer sensor of the collar device was able to effectively detect and measure the motion patterns in the three-axis. The sensor could identify and classify specific behaviour categories, such as grazing, and rumination based on patterns of head movements. The behaviour data collected by the sensor were analysed by the collar device using built-in generic algorithms and produced as minute-per-hour (min/h) behaviour counts (per-hour grazing time and rumination time). The data collected by the AfiCollar device were wirelessly transmitted to a base station through Wi-Fi while cows were in the range of ~500 m. The data were manually downloaded from the computer attached to the base station in a Microsoft Excel spreadsheet (Microsoft Excel, version 2016). The minute-per-hour behaviour counts per daily observation session (8 h) by the collar device were used to manually calculate total grazing time and rumination time per daily observation session.

Each cow being observed was fitted with an AfiCollar device around the neck, which was worn by the cow throughout the observation period. Each collar device was time synchronised and activated by Afimilk’s herd management software (Afimilk mySilent Herdsman, Afimilk Ltd. Kibbutz Afikim 1514800, Israel) before being placed on the experimental animals. The AfiCollar device continuously monitored and recorded the time spent by the cows on grazing and ruminating on a real-time basis.

### 2.4. Data Preparation and Statistical Analysis

A total of 144 observations (48 cows × 3 daily observation sessions per cow) were separately collected through both AfiCollar and visual observation over the observation period. The data recorded through the AfiCollar device and the data obtained through visual observation were coupled with each other. Both recorded and observed outputs were presented as total minutes each cow spent on grazing and rumination activities per daily observation session.

To investigate the levels of correlation and agreement between observed and recorded outputs, Pearson’s correlation coefficient (r) and concordance correlation coefficient (CCC) were calculated respectively for both grazing time and rumination time as indicated by Laurance and Kuei [34]. The values of r were calculated using SAS (version 9.4) and the value of CCC was calculated following the equation as suggested by Laurance and Kuei [35]. The interpretations of r and CCC were classified as: negligible = 0.0–0.3, low = 0.3–0.5, moderate = 0.5–0.7, high = 0.7–0.9, and very high = 0.9–1.00 [35]. Mean bias (MB) was also calculated to examine the mean difference in grazing time and rumination time between the AfiCollar device and visual observation. Relative prediction error (RPE) values were calculated following Stergiadis [36] to evaluate the accuracy of the AfiCollar device to record grazing time and rumination time using the following equation [37]:

$$RPE=\left(\frac{\sqrt{MPSE}}{A𝓂}\right)\times 100$$

where MPSE is the mean square prediction error and A𝓂 is the mean of the observed counts for each behaviour.

To further investigate the associations between visual observation and automatic recording, linear regression analysis was performed in SAS (version 9.4) with R-squared values reported for both grazing time and rumination time [38].

## 3. Results

### 3.1. Comparison of the AfiCollar and Visual Observation

#### 3.1.1. Grazing Behaviour

**Figure 1:**
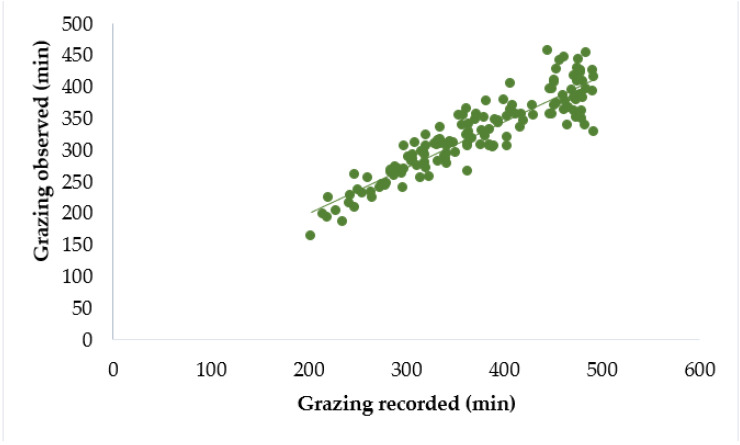
Relationship between grazing time recorded through AfiCollar device and grazing time obtained through visual observation per daily observation session.

The comparison was made between the behaviour results recorded through AfiCollar (AC) and the results obtained through visual observation (VO) and details are presented in Table 2. The mean time spent grazing per cow per daily observation session recorded through AC was 373 min while that obtained through VO was 325 min. The mean bias of grazing time between AC results and VO results was 48 min. Compared to VO results, AC results showed higher grazing time (10% on average). AC results and VO results had a good association (Figure 1), showing a strong correlation and high level of agreement (r = 0.91, CCC = 0.71) between each other with an RPE value of 0.14. Regression analysis (Table 3) showed, VO result had a significant linear relationship with AC results (R^2^ = 0.83, *p* < 0.05). The intercept and slope for grazing time were significantly different from zero (*p* < 0.05).

#### 3.1.2. Rumination Behaviour

**Figure 2:**
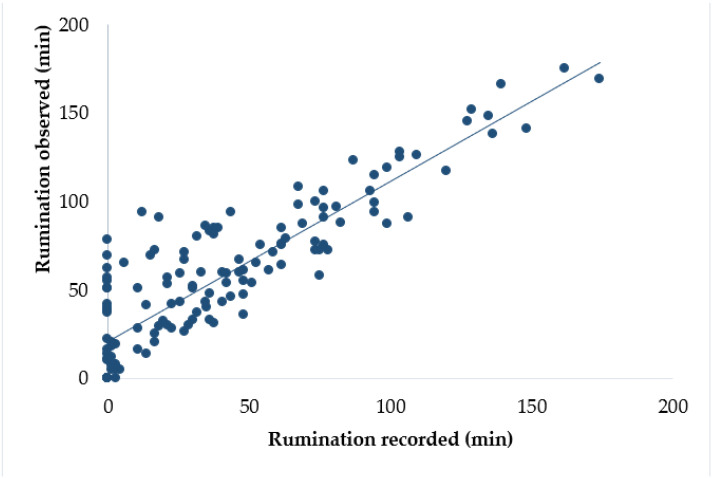
Relationship between rumination time recorded through the AfiCollar device and rumination time obtained through visual observation per daily observation session.

The comparison was made between the behaviour results recorded through AC and results obtained through VO and details are shown in Table 2. The mean rumination time per cow, per daily observation session recorded through AC, was 39 min, whereas that obtained through VO was 56 min. Compared to the VO results, AC results showed lower rumination time (4% on average). The mean bias of rumination time per daily observation session calculated between two methods was 17 min. The AC results and VO results had a good association (Figure 2), showing strong correlation and level agreement (r = 0.89, CCC = 0.80) between each other with an RPE value of 0.40. Some of the cows (*n* = 15) were not found ruminating both in AC and VO results during some of the daily observation sessions. Moreover, for some of the observation sessions, rumination activity was obtained through VO, but not produced by AC results. Regression analysis (Table 3) showed that the VO results had a significant linear relationship with the AC results (R^2^ = 0.78, *p* < 0.05). The intercept and slope for rumination time were significantly different from zero (*p* < 0.05).

## 4. Discussion

This study validated the accuracy of a triaxial accelerometer sensor-based automated device called AfiCollar, designed to monitor and record eating and rumination behaviours on a real-time basis. The collar device was earlier tested in an indoor dairy system [30] and reported to have 87% accuracy in recording the eating and rumination behaviours of dairy cows. However, in the present study, the collar device was evaluated to monitor and record grazing and rumination behaviours of dairy cows in a pasture-based system in a New Zealand-based farm.

Consistent behaviour counts were found between automatic recording through the AfiCollar (AC) and visual observation (VO) throughout the observation period. The estimation of mean grazing time per daily observation session through AC was slightly higher than that of VO, with a mean bias of 48 min (10%). In a grazing-based system, dairy cows have freedom of movement, and they keep moving between the feeding patches in search of quality feed [39]. Therefore, the frequency of movements in a grazing-based system is higher than that in a confined indoor system. Moreover, during performing the visual observation for the current study, cows were found with a high frequency of movements, spending more time selecting grazing patches and moving around without performing grazing activity. The AfiCollar device is equipped with an accelerometer-based sensor that is designed to detect motion patterns. The sensor can identify and categorise specific behaviour categories based on the motion patterns of animal’s head. The accelerometer-based sensor in the collar device possibly considered all those movements by the cows with their heads down to select grazing patches as true grazing activity. The built-in algorithms in the collar device possibly calculated the time spent on those movements with no grazing activity by cows as grazing time. The higher grazing time recorded by AC compared to VO was probably due to the interpretation of those false positive grazing movements as true grazing activity.

For the rumination behaviour, a lower rumination time was recorded through AC than that indicated by VO, with a mean bias of 17 min (4%). Rumination time between AC and VO mainly differed in the recording performed during the hot summer season. Cows were expected to be feeling heat stress as they were observed performing rumination activity mostly in a standing posture. It was noted previously, as temperature increases in hot summer, cows stand more and lie down less [40]. Moreover, pastured cows are exposed to fly attacks during the summer, exacerbated by increased heat, as reported in a previous study [41]. The cows were also observed frequently performing random head movements to get rid of flies around the lower abdomen and udder areas that are common on pasture in the summer season. The collar uses an accelerometer-based sensor that might have considered and interpreted those random head movements performed by the cows during the standing posture as grazing activity, whereas the cow was performing rumination. The sudden and frequent random head movement probably would have interrupted the threshold of specific movement patterns for rumination behaviour identified by the sensor. Afterwards, the collar might have started counting the head movements into grazing time. These findings further suggest that the algorithms used by the collar could be improved in terms of precision to truly identify and interpret the patterns of head movements reflecting specific behaviour activity. Some cows (*n* = 15) were found with no rumination activity in both AC and VO results during some of the observation days. As previously reported, the maximum rumination activity by dairy cows is performed during the night while resting [42]. This might be the possible reason for zero rumination during the observation period, which was during the daytime (from 9:00 a.m. to 5:00 p.m.). On the other hand, some cows were recorded with zero or no rumination activity, but were observed performing rumination activity. This occurred during the observation performed in the summer season as explained above. This might be because of atmospheric patterns and high-temperature humidity index, which affected the behaviour of dairy cows. The behaviour variation due to high temperature was reflected in the outputs recorded through AC. This will be further investigated.

The strong correlation between AC results and VO results for grazing (r = 0.91) and rumination (r = 0.89) indicated high accuracy of the collar to monitor grazing and rumination behaviours of grazing dairy cows. The correlation values were consistent with the findings of previous studies which used accelerometer sensor-based collars. A recent study [28] reported the correlation values for grazing (r = 0.88) and rumination (r = 0.72) between observed and recorded results using an ear-tag accelerometer-based sensor (Cow Manager Sensor, Agis Automatisering BV, Harmelen, the Netherlands), which identified the specific behaviour based on both ear and head movements. Another study reported a high correlation between observed and recorded results for grazing behaviour (r = 0.90) and rumination behaviour (r = 0.80) in grazing dairy cows [3]. The device used in that study was a neck-mounted behaviour and activity monitoring collar (SCR HR-LDn; SCR Engineers, Netanya, Israel), which had a three-axis accelerometer sensor to generate data and, a microprocessor to calculate (utilizing specifically developed algorithms) grazing and rumination behaviours. The consistent correlation values between visual observation and automatic measurements in the current study with that of previous studies suggested the reliability of the AfiCollar device to effectively monitor grazing and rumination behaviours of grazing dairy cows.

Concordance correlation coefficient (CCC) values between AC and VO were high for both grazing (CCC = 0.71) and rumination (CCC = 0.80). However, the value for rumination was higher than that of grazing. Those values based on the agreement between recorded and observed datasets further explained the reliability of the collar to accurately record grazing and rumination behaviours of grazing dairy cows. The CCC values for grazing and rumination agreed with the CCC values reported by previous studies. A study [32] reported a slightly higher CCC value for grazing (CCC = 0.88) and a slightly lower CCC value for rumination (CCC = 0.71) compared to CCC values in the current study. Another study used an accelerometer sensor-based device called CowManager SensOor (Agis, Harmelen, Netherlands) and reported CCC = 0.82 for eating and CCC = 0.59 for rumination in dairy cows [43]. Moreover, the relative prediction error for rumination (0.40) was higher than that for grazing (0.18). This indicated that the precision of the collar device for monitoring grazing behaviour was higher than that of rumination behaviour.

Regression analysis further verified a strong relationship between observed and recorded results for both grazing and rumination. High R^2^ values were found between AC results and VO results in the current study for grazing (0.83) and rumination (0.78). High R^2^ values indicated the efficiency of the collar to monitor and record behaviours in a grazing-based system. A previous study [44] found a range of R^2^ values from 0.69 to 0.93 between recorded and observed results for the grazing behaviour of dairy cows. They used Kenz Lifecorder+^®^ (LC+; Suzuken Co. Ltd., Nagoya, Japan) which was developed to monitor uniaxial acceleration. Similarly, another study has found R^2^ value (0.79) for rumination consistent with the value in the current study [45]. A range of R^2^ values (from 0.22 to 0.79) between recorded and observed data for the rumination behaviour in different aged day cows has been reported [39]. Their study used the Hi-Tag electronic rumination-monitoring system (SCR Engineers Ltd., Netanya, Israel). Highly consistent with the previous studies, R^2^ values for both behaviours in the current study further proved the reliability and accuracy of the AfiCollar to monitor the behaviour of grazing dairy cows.

## 5. Conclusions

This study evaluated a triaxial accelerometer sensor-based automated device (AfiCollar) for dairy cows to monitor and record their grazing and rumination behaviours in a grazing-based system on a real-time basis. The AfiCollar device showed a strong correlation and high agreement with visual observation. Based on associations between automatic recording and visual observation, the AfiCollar device proved to be a useful and reliable tool to accurately monitor and record the grazing and rumination behaviours of grazing dairy cows on a pasture on a real-time basis. The collar slightly overestimated grazing time and underestimated rumination time, although the difference was not substantial. This was possibly because of the effects of the weather (high temperature) on the behaviour of dairy cows, which was reflected in behaviour outputs by the collar device. Minor modifications are suggested in algorithms to improve the identification and characterization of cow head movements for a more precise interpretation of the specific behaviour.

## Figures and Tables

**Table 1:** Time of year for the visual observation and their duration, and behaviour activities observed during the recording periods.

Recording Period	Duration		Observation Sessions	Behaviour Observed
	From	To		
1	19 December 2019	28 February 2020	9 (72 h)	Grazing and rumination
2	30 September 2020	16 October 2020	9 (72 h)	Grazing and rumination
3	2 December 2020	18 December 2020	9 (72 h)	Grazing and rumination
4	8 February 2021	5 March 2021	9 (72 h)	Grazing and rumination

(Note: each observation session consisted of 8 h per daily recording, so, 9 × 8 = 72).

**Table 2:** Comparison of grazing time and rumination time, mean bias (MB), percentage mean bias, Pearson’s correlation coefficient (r), concordance correlation coefficient (CCC), and relative prediction error (RPE) of behaviour data recorded by AfiCollar (AC) and collected with visual observation (VO). (*n* = 144).

Behaviour	AC (min)	VO (min)	MB (min)	Bias ^1^ (%)	r	CCC	RPE
Grazing time	373 ± 78.9	325 ± 62.9	48	10	0.91	0.72	0.18
Rumination time	39 ± 40.9	56 ± 42.1	17	04	0.89	0.80	0.40

^1^ Bias (%) represents mean percentage bias and it was calculated as: AC (min)/480 − VO (min)/480 × 100, where 480 is the total time (8 h.) per daily observation session.

**Table 3:** Regression analysis results between the AfiCollar and visual observation for grazing time and rumination time.

	Grazing Time	Rumination Time
R^2^	0.83 (*p* < 0.0001)	0.78 (*p* < 0.0001)
Slope (SEM, *p*)	0.72 (0.02, *p* < 0.0001)	0.90 (0.04, *p* < 0.0001)
Intercept (SEM, *p*)	54.3 (10.5, *p* < 0.0001)	20.9 (2.31, *p* < 0.0001)

(The regression model between visual observation (on *Y*-axis) and AfiCollar (*X*-axis) measurements (min/observation) are presented with the coefficients of determination (R^2^), the slops, and the intercepts with standard errors of the mean (SEM) and *p*-value. The significance level for *p*-value was set at 0.05).
